# Higher Body Mass Index Is Related to Severe Chemotherapy-Induced Peripheral Neuropathy in Patients with Ovarian Cancer: A Preliminary Retrospective Study

**DOI:** 10.3390/jcm14134485

**Published:** 2025-06-25

**Authors:** Jin-Mo Park, Incheol Seo, Minsung Kang, Gun Oh Chong, Yoon Hee Lee, Jin-Sung Park

**Affiliations:** 1 Department of Neurology, Dongguk University College of Medicine, Dongguk University Gyeongju Hospital, Gyeongju 38067, Republic of Korea; 2 Department of Immunology, School of Medicine, Kyungpook National University, Daegu 41944, Republic of Korea; 3 Department of Neurology, Kyungpook National University Chilgok Hospital, Daegu 41404, Republic of Korea; 4 Department of Obstetrics and Gynecology, School of Medicine, Kyungpook National University, Kyungpook National University Chilgok Hospital, Daegu 41404, Republic of Korea; 5 Department of Neurology, School of Medicine, Kyungpook National University, Kyungpook National University Chilgok Hospital, Daegu 41404, Republic of Korea

**Keywords:** chemotherapy-induced peripheral neuropathy, ovarian cancer, risk factor, paclitaxel, carboplatin, body mass index, neuropathy scale

## Abstract

**Background:** Chemotherapy-induced peripheral neuropathy (CIPN) is a debilitating side effect of cancer treatment, which is more common in patients with ovarian cancer who are receiving paclitaxel and carboplatin treatment. Although prior studies have explored the association between obesity and CIPN, most relied on subjective grading systems. This study aims to evaluate the relationship between being overweight and CIPN using the Total Neuropathy Score (TNS), a more objective and comprehensive assessment tool. The purpose of this study is to investigate the relationship between being overweight and the onset and severity of CIPN in a cohort of South Korean patients with ovarian cancer who were treated with paclitaxel and carboplatin. **Methods:** The study was conducted retrospectively at a single tertiary hospital in South Korea from March 2013 to February 2022. Included in this study were forty-two individuals who were diagnosed with epithelial ovarian cancer and who had developed neuropathic symptoms following chemotherapy. Patient characteristics, laboratory findings, and disease-specific TNS were analyzed. **Results:** Overweight patients showed significantly more severe CIPN, with higher Total Neuropathy Scores (TNS; *p* = 0.019) and earlier symptom onset (*p* < 0.05) compared to normal-weight patients. Causal mediation analysis also revealed a significant direct effect of BMI on TNS (*p* = 0.006). However, no significant correlation was found between BMI and TNS using Kendall’s rank correlation, and other neuropathic symptoms or laboratory parameters did not show statistically significant group differences. **Conclusions:** The study suggests that overweight may be associated with the severity and onset of CIPN in patients with ovarian cancer who are receiving paclitaxel and carboplatin chemotherapy. While the results are preliminary, they underscore the importance of addressing this modifiable risk factor in clinical care. Further research is needed to better understand the underlying mechanisms and to inform future therapeutic strategies.

## 1. Introduction

Chemotherapy-induced peripheral neuropathy (CIPN) is a debilitating adverse effect of chemotherapy treatments, such as carboplatin and paclitaxel, which are commonly used to treat ovarian cancer [1,2]. Due to its potential to cause irreversible functional impairment and decreased quality of life, CIPN remains a significant challenge in the management of patients with cancer despite its high prevalence [3,4]. There is a paucity of effective prevention and treatment strategies available for CIPN. Currently, the only recommended treatment to mitigate CIPN is dose modification, but this approach may reduce treatment efficacy, and its impact on long-term CIPN outcomes remains poorly characterized [5,6]. Although the causes of CIPN are not well understood, patient characteristics, comorbidities, and lifestyle have been indicated as potential risk factors [7]. Obesity is one condition of particular interest, which is a prevalent condition associated with metabolic syndrome and has been proposed as a potential risk factor for CIPN [7,8,9]. Central obesity has been associated with a higher incidence of neuropathy in non-cancer populations, implying a link to neurotoxic effects [10,11]. Nonetheless, given the contradictory findings seen in prior research, the relationship of obesity to the development of CIPN remains unclear [12,13]. This could be explained by variations in the demographics of study participants, the timing of evaluations, and the criteria employed for defining CIPN.

The purpose of this study is to evaluate the association between being overweight and the developmental and functional outcomes of CIPN in patients with ovarian cancer in South Korea who received paclitaxel and carboplatin treatment.

## 2. Methods

### 2.1. Participants

This study was conducted retrospectively at a single tertiary hospital in South Korea from 2013 to 2022. The study population consisted of patients who were diagnosed with epithelial ovarian cancer and who had experienced neuropathic symptoms after the initiation of chemotherapy with paclitaxel and carboplatin. It included patients diagnosed with epithelial ovarian cancer who experienced neuropathic symptoms after starting chemotherapy with paclitaxel and carboplatin. Patients had to be 18 years or older and have completed chemotherapy 3 to 12 months prior to the study. The study focused on those who had undergone either primary surgical debulking or interval debulking following three cycles of neoadjuvant chemotherapy. Patients with a medical history of renal or hepatic failure or with preexisting peripheral neuropathy from diabetes or any other etiology were excluded from the study.

The participants in this study were subjected to the following chemotherapy regimen: Paclitaxel (175 mg/m^2^ of body surface area (BSA)) was given intravenously over 3 h as part of the chemotherapy regimen. This was followed by carboplatin (area under the curve 5) over 1 h, with standard antiemetic and hypersensitivity medications. All patients were treated with the 3-weekly regimen that consisted of a 21-day cycle repeated for 6 cycles followed by either three or six cycles based on clinical response.

Due to its retrospective nature, this study received a waiver from the need for individual patient consent and was approved by the Institutional Review Board of Kyungpook National University Chilgok Hospital (KNUCH 2023–09-004).

### 2.2. Clinical and Laboratory Evaluations

At the time chemotherapy was started, data on height, weight, body mass index (BMI), and existing underlying conditions such as hypertension and diabetes were extracted from medical records. BMI was ascertained by dividing weight (in kilograms) by the square of height (in meters), categorizing patients as normal weight (BMI < 25 kg/m^2^) or overweight (BMI ≥ 25 kg/m^2^). The BSA was calculated by using the Mosteller equation, which guided the chemotherapy dosage recommendations. Additionally, a number of laboratory parameters were collected from the patient’s medical records. The results of blood tests that were conducted before the start of chemotherapy treatment are the following: Albumin, erythrocyte sedimentation rate (ESR), hemoglobin, homocysteine, ionized calcium, ionized magnesium, methylmalonic acid, C-reactive protein (CRP), creatinine, vitamin B12, vitamin D, aspartate transaminase (AST), alanine transaminase (ALT), ferritin, cancer antigen 125 (CA-125), carcinoembryonic antigen (CEA), carbohydrate antigen 19.9 (CA-19.9), and absolute neutrophil count (ANC).

### 2.3. Neuropathy Assessment

All patients presenting with neurological symptoms were examined by experienced neurologists with expertise in neuromuscular disease. The “time to onset of peripheral neuropathy” was calculated from the start of chemotherapy to the first instance of reported neuropathic symptoms. These symptoms include sensory abnormalities such as numbness or tingling, burning sensations, and pain. Additionally, patients might experience weakness, along with difficulties in coordination or balance. These manifestations typically occur in a ‘glove and stocking’ pattern, predominantly affecting the hands and feet. Four specific aspects of sensory impairment, including allodynia, hyporeflexia, large fiber deficit, and paresthesia, were the focus of the neurologic examination. Nerve conduction studies involving the median, ulnar, peroneal, tibial, superficial peroneal, sural, and medial plantar nerves were completed to confirm the presence of large fiber neuropathy. Based on the results of these tests, the total neuropathy score (TNS) was calculated. TNS is a widely used clinical tool to evaluate CIPN, and it assesses the degree of sensory loss, muscle weakness, deep tendon reflexes, and autonomic dysfunction [14]. The TNS has a range of 0 to 136, and it correlates well with the objective severity of the neuropathy in CIPN. Higher scores indicate greater severity and extent of neuropathy. These neurologic evaluations are conducted within a few days of the patients presenting at the neurology department.

### 2.4. Statistical Analysis

Differences in patient characteristics, laboratory test results, and neurological examination findings between BMI groups were assessed using independent *t*-tests or Mann–Whitney U tests for continuous variables, depending on data normality, and Fisher’s exact tests for categorical variables. Normality of continuous variables was evaluated with the Shapiro–Wilk test to determine the appropriate statistical test. *p*-values from multiple comparisons were adjusted using the Benjamini–Hochberg (BH) method to control the false discovery rate. A *p*-value of less than 0.05 was considered statistically significant.

Additionally, Kendall’s rank correlation analysis was performed to evaluate associations among variables. Post hoc power analysis was conducted for neurological symptoms by BMI group to assess statistical power. Furthermore, causal mediation analysis was performed to investigate the role of body surface area (BSA) in the relationship between BMI and chemotherapy-induced peripheral neuropathy (CIPN) severity. All analyses were performed using R (version 4.2.2).

## 3. Results

### 3.1. Patient Characteristics

A total of 615 patients underwent surgery for ovarian cancer at our institution. Among them, 528 patients received chemotherapy. This study focused on forty-two of these patients who had epithelial ovarian cancer, received paclitaxel and carboplatin treatment, and subsequently developed neuropathic symptoms. Of these patients, 24 were classified as having normal weight, and 18 were classified as overweight. Table 1 shows the baseline clinical characteristics of the 42 patients. The mean age of the normal weight group was 58.60 ± 8.04 years, while the mean age of the overweight group was 59.38 ± 7.83 years. There were 5 (11.90%) patients in stage I, 5 (11.90%) in stage II, 25 (59.52%) in stage III, and 7 (16.67%) in stage IV. Age, cancer stage distribution, and comorbidity rates—type 2 diabetes (16.7% vs. 16.7%, *p* = 0.118) and hypertension (26.2% vs. 26.2%, *p* = 0.159)—did not differ significantly between the normal-weight and overweight groups. The mean body mass index of the normal weight group was 24.70 ± 3.52 kg/m^2^, while the mean BMI of the overweight group was 28.01 ± 2.65 kg/m^2^. The mean BSA of the normal-weight group was 1.51 ± 0.10 m^2^, while the mean BSA of the overweight group was 1.71 ± 0.12 m^2^. The difference in mean BMI and BSA between the two groups was statistically significant (*p* < 0.001). However, the mean cumulative doses of paclitaxel and carboplatin were not significantly different between the normal weight and the overweight groups.

### 3.2. Laboratory Findings

Significant relationships between vitamin B12 and ESR were revealed in clinical parameter analyses (τ = 0.35, *p* < 0.001), as well as between vitamin D levels (τ = 0.3, *p* < 0.001) (Supplementary Figure S1).

In relation to laboratory findings, there were no significant differences between the normal-weight and overweight cohorts. The mean hemoglobin, inflammatory markers (ESR, CRP), albumin (reflecting nutritional status), vitamin levels, and tumor markers (CA-125, CEA, CA 19-9) did not differ significantly between the two groups (Table 2).

### 3.3. Variables Related to the Severity of CIPN

We explored the correlation between the TNS and various laboratory findings (Supplementary Table S2). Levels of vitamin B12, methylmalonic acid, and homocysteine showed no significant association with TNS. Similarly, other laboratory markers, including CRP and hemoglobin, were not correlated with TNS. Only CEA demonstrated a statistically significant positive correlation with TNS (τ = 0.38, *p* = 0.013). None of the demographic characteristics showed a significant correlation with TNS (Table 3).

### 3.4. Impact of BMI on Neuropathy Severity and Onset

**Figure 1:**
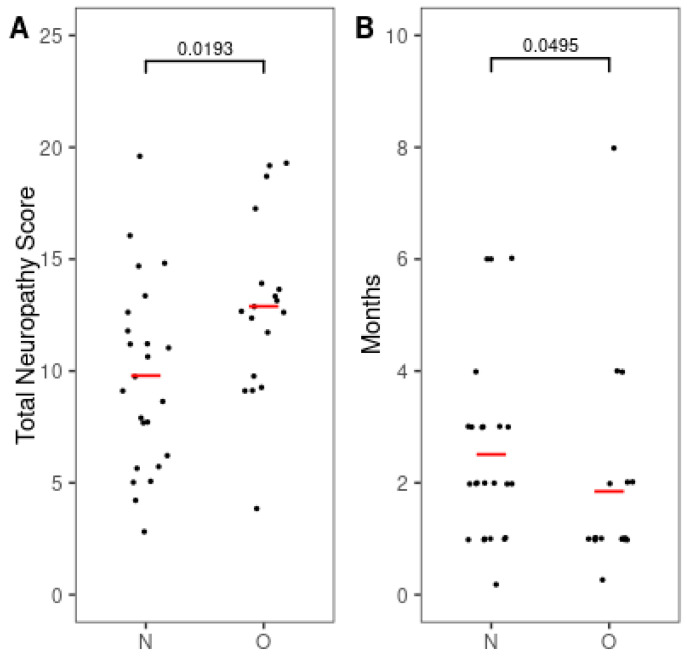
Differences in severity and onset interval of chemotherapy-induced peripheral neuropathy by the BMI group. (**A**) Total neuropathy score (TNS). (**B**) Time to onset of peripheral neuropathy after chemotherapy. Red bars indicate mean values. Numbers indicate *p*-values. *p*-values were calculated using the Mann–Whitney U test for TNS and the independent *t*-test for time to onset. N, normal weight group; O, overweight group.

Given that BMI was not normally distributed in our sample (Table 1) and the limited sample size may reduce power to detect subtle effects, we compared the severity and onset of CIPN between BMI groups. The TNS was significantly higher in the overweight group (12.89 ± 3.95) compared to the normal weight group (9.79 ± 4.21; *p* = 0.019; Figure 1A), indicating more severe neuropathy.

Although cumulative paclitaxel and carboplatin doses did not differ between BMI groups, BSA-based dosing in overweight patients may result in higher absolute drug exposure. To investigate whether this pharmacokinetic mechanism contributes to CIPN, we conducted a causal mediation analysis evaluating BSA as a potential mediator in the relationship between BMI and CIPN severity (measured by TNS; Supplementary Table S2). The analysis revealed a statistically significant direct effect of BMI on CIPN severity (*p* = 0.006), indicating that higher BMI is associated with more severe neuropathy. However, the indirect effect through BSA was not statistically significant, suggesting that BSA does not significantly mediate the relationship between BMI and CIPN severity in this sample. While the hypothesized pathway of BMI → BSA → higher drug exposure → CIPN remains biologically plausible, these results do not support a significant mediating role of BSA in this context.

Furthermore, the onset of CIPN occurred significantly earlier in the overweight group (1.85 ± 1.84 months) than in the normal weight group (2.51 ± 1.63 months; *p* < 0.05; Figure 1B).

### 3.5. Neurological Examination Findings

We evaluated neuropathic symptoms based on allodynia, hyporeflexia, large fiber deficit, or paresthesia (Table 4). No statistically significant differences were observed between normal-weight and overweight patients in these neuropathic symptoms. Post hoc power analyses were conducted for the neurological outcomes to assess whether the study was adequately powered to detect the observed differences between BMI groups.

The comparison for large fiber deficit yielded the largest effect size (h = 0.770) and the highest calculated power (69.4%), suggesting moderate power to detect a difference in this outcome. However, the comparisons for hyporeflexia (h = 0.365; power = 21.6%), paresthesia (h = 0.472; power = 32.8%), and allodynia (h = 0.110; power = 6.4%) all showed low statistical power.

## 4. Discussion

We investigated the potential association between obesity and the development of CIPN in patients with ovarian cancer who received paclitaxel and carboplatin treatment. To identify the possible laboratory risk factors that may lead to CIPN, we retrospectively assessed the laboratory findings before treatment. Interestingly, no significant laboratory results were found that could account for the higher risk of CIPN in our study. Previous studies have illustrated that low hemoglobin or abnormality in albumin are potent risk factors of CIPN [7,15,16,17]. The pathomechanism of anemia is still poorly understood, but after treating anemia in patients with peripheral neuropathy, they showed significant improvement in a number of patients with cancer [7]. Conflicting findings exist regarding albumin levels, with some studies suggesting higher albumin as a risk factor due to oxidative stress and inflammation [15,16], while others suggest low albumin levels as a risk factor due to malnutrition [15]. These controversial results are in line with our study, which also failed to identify a significant correlation between hemoglobin or albumin levels and TNS, further emphasizing the need to look beyond conventional laboratory markers for CIPN risk prediction. Hypocalcemia was identified as a risk factor for CIPN in a study on taxane-induced peripheral neuropathy in patients with breast cancer [8]. However, in our study, we were unable to confirm any association between calcium levels and TNS (τ = 0.03, *p* = 0.759). Moreover, our analysis of laboratory results revealed no significant differences in various parameters, including albumin, ESR, hemoglobin, homocysteine, ionized calcium, ionized magnesium, methylmalonic acid, CRP, creatinine, vitamin B12, AST, ALT, ferritin, CA-125, CA-19.9, and ANC, between patients with normal weight and those who are overweight. This implies that the differences observed in the occurrence of CIPN cannot be attributed to these laboratory factors.

We additionally conducted a causal mediation analysis to investigate whether BSA mediates the relationship between BMI and CIPN severity. However, the indirect effect through BSA was not statistically significant, suggesting that BSA does not play a mediating role in this association in our sample.

Another factor that may have influenced our findings is the selection pathway of patients. Among the 528 patients who received chemotherapy for ovarian cancer, only 42 were diagnosed with CIPN after visiting a neurology clinic. This low number suggests that CIPN might be underdiagnosed in this population. Several factors could contribute to this underestimation. Firstly, patients undergoing cancer treatment often prioritize their therapy over managing side effects like neuropathic symptoms, especially if these symptoms are not severe [18]. As a result, patients with milder symptoms may not seek neurological evaluation, leading to their exclusion from our cohort. This referral pattern likely resulted in a sample skewed toward more symptomatic or severe cases, which may have contributed to an overestimation of CIPN severity—particularly in overweight patients. Secondly, CIPN symptoms can be variable and sometimes mild, leading patients to underreport them. This variability in symptom presentation can make it challenging to diagnose CIPN without a thorough neurological evaluation. Additionally, some patients might not be aware that their symptoms are related to their chemotherapy, further contributing to underreporting [19]. Thirdly, there may be a lack of routine screening for CIPN in oncology settings. Without standardized protocols for monitoring and diagnosing neuropathic symptoms, many cases might go undetected until they become more severe [20].

We acknowledge several limitations and strengths of this study. Its retrospective design and limited sample size may have restricted the generalizability and statistical power of our findings. Additionally, since most participants were recruited from our neurology clinic, the sample may not fully represent the broader population of ovarian cancer patients undergoing chemotherapy. As indicated by our post hoc power analysis of neurological symptoms by BMI group, the study was underpowered to detect certain differences, limiting our ability to control for potential confounders such as age, diabetes mellitus, and hypertension. However, univariate linear regression analyses did not show statistically significant effects of these variables on TNS or CIPN onset time (Supplementary Table S3). Moreover, the sample distribution across demographic variables was uneven. Although no statistically significant differences were found, larger and more balanced samples are needed to improve statistical robustness. Our sample size was also insufficient to explore the effect of BMI as a continuous variable on CIPN severity or onset time or to divide participants into normal weight, overweight (25–29.9 kg/m^2^), and obese (≥30 kg/m^2^) categories. Consequently, we compared only normal-weight versus overweight groups, limiting the granularity of our analysis. Additionally, although large-fiber deficit showed moderate power (69.4%), power for allodynia, hyporeflexia, and paresthesia was low (6.4–32.8%), indicating that some nonsignificant results may reflect insufficient power rather than a true absence of effect. Thus, the possibility of Type II errors must be considered. Moreover, because our study focused on CIPN onset within 3 to 12 months post-chemotherapy, we were unable to evaluate long-term outcomes such as symptom persistence or recovery patterns. The lack of longitudinal follow-up data limits our ability to draw conclusions about the chronicity or reversibility of CIPN, which are critical for informing long-term patient management. Finally, due to the retrospective design of our study, causal relationships cannot be definitively determined. Future studies with larger, well-powered cohorts are needed to better assess these associations.

Despite these limitations, our study has several notable strengths. First, we employed the full TNS, which includes both clinical assessments and electrophysiological evaluations, allowing for a more objective and comprehensive evaluation of CIPN. This contrasts with prior studies that primarily used subjective tools such as the Common Terminology Criteria for Adverse Events, the FACT/GOG-NTX, or the clinical TNS (TNSc), which omit nerve conduction studies and quantitative sensory testing [7,8,9]. Full TNS provides enhanced diagnostic accuracy by assessing multiple domains, including autonomic dysfunction [21]. Second, our study adds to the scarce data from East Asian populations—specifically South Korean women with ovarian cancer. Most previous research linking BMI and CIPN has focused on Western breast cancer cohorts, limiting the applicability of findings across ethnic and disease groups [7,8,9,22].

## 5. Conclusions

In conclusion, our study provides preliminary evidence of an association between higher BMI and increased risk and severity of CIPN among patients with ovarian cancer treated with paclitaxel and carboplatin. While the underlying mechanisms remain incompletely understood, these findings highlight the importance of considering obesity—a modifiable risk factor—in the clinical management of ovarian cancer to reduce the burden of CIPN. Further well-powered prospective studies are warranted to clarify this relationship and inform more effective prevention and treatment strategies.

## Figures and Tables

**Table 1:** Baseline characteristics of the study population.

Characteristics	Total n = 42	Normal Weight n = 24	Overweight n = 18	*p*-Value
Age (mean, SD)	58.60 (8.04)	59.38 (7.83)	57.56 (8.42)	0.480
Cancer stage (n, %)				0.523
I	5 (11.90%)	3 (12.50%)	2 (11.11%)	
II	5 (11.90%)	4 (16.67%)	1 (5.56%)	
III	25 (59.52%)	12 (50.00%)	13 (72.22%)	
IV	7 (16.67%)	5 (20.83%)	2 (11.11%)	
Type 2 DM (n, %)	7 (16.67%)	2 (8.33%)	5 (27.78%)	0.118
Hypertension (n, %)	11 (26.19%)	4 (16.67%)	7 (38.89%)	0.159
BSA (mean, SD)	1.60 (0.15)	1.51 (0.10)	1.71 (0.12)	<0.001
BMI * (mean, SD)	24.70 (3.52)	22.21 (1.38)	28.01 (2.65)	<0.001
Cumulative dose (mean, SD)				
Paclitaxel * (mg)	1990.93 (798.95)	1917.853 (620.55)	2088.366 (1000.44)	0.554
Carboplatin (mg)	4255.377 (1737.62)	4157.943 (1446.03)	4385.289 (2102.78)	0.696

*p*-values were calculated using an independent *t*-test or the Mann–Whitney U test * for continuous variables and Fisher’s exact test for categorical variables. SD, standard deviation; DM, diabetes mellitus; BSA, body surface area (m^2^); BMI, body mass index.

**Table 2:** Laboratory findings of the study population.

Characteristics (mean, SD)	Total n = 42	Normal Weight n = 24	Overweight n = 18	*p*-Value
Albumin (g/dL)	4.19 (0.34)	4.18 (0.36)	4.21 (0.33)	0.867
ESR (mm/h)	37.57 (26.27)	33.92 (25.77)	42.44 (26.87)	0.309
Hemoglobin * (g/dL)	11.78 (1.51)	11.73 (1.23)	11.84 (1.86)	0.836
Homocysteine (μmol/L)	8.80 (2.39)	8.55 (2.50)	9.13 (2.26)	0.394
Ionized calcium * (nmol/L)	1.22 (0.05)	1.21 (0.06)	1.23 (0.04)	0.243
Ionized magnesium * (mmol/L)	0.54 (0.07)	0.56 (0.06)	0.53 (0.09)	0.202
Methylmalonic acid (μmol/L)	0.24 (0.20)	0.25 (0.24)	0.22 (0.12)	0.939
CRP (mg/L)	2.65 (3.72)	3.26 (4.43)	1.83 (2.36)	0.292
Creatinine (mg/dL)	0.70 (0.18)	0.67 (0.14)	0.75 (0.21)	0.258
Vitamin B12 (pg/mL)	853.83 (382.60)	813.79 (357.81)	907.22 (417.80)	0.542
Vitamin D (nmol/L)	39.63 (63.34)	33.01 (43.92)	48.97 (84.26)	0.348
AST * (U/L)	25.55 (6.37)	26.04 (6.88)	24.89 (5.74)	0.558
ALT (U/L)	21.21 (8.17)	21.25 (8.24)	21.17 (8.32)	0.819
Ferritin (μg/L)	141.58 (119.97)	157.41 (148.20)	124.75 (81.80)	0.627
CA-125 (U/mL)	1053.70 (2107.35)	1227.80 (2663.48)	831.23 (1084.06)	0.351
CEA (μg/L)	42.26 (199.57)	63.56 (249.72)	4.41 (6.69)	0.469
CA 19.9 (U/mL)	94.36 (210.37)	63.40 (189.93)	131.51 (237.41)	0.497
ANC	2900.06 (1864.87)	2643.44 (1127.74)	3242.22 (2539.67)	0.675

*p*-values were calculated using an independent *t*-test * and Mann–Whitney U test for normally and non-normally distributed data, respectively. SD, standard deviation; ESR, erythrocyte sedimentation rate; CRP, C-reactive protein; AST, aspartate transaminase; ALT, alanine transaminase; CA-125, cancer antigen 125; CEA, carcinoembryonic antigen; CA 19.9, carbohydrate antigen 19.9; ANC, absolute neutrophil count.

**Table 3:** Correlation between demographic characteristics and Total Neuropathy Score (TNS).

Characteristics	Kendall’s Rank Correlation Coefficient (τ) with TNS	*p*-Value
Age	0.16	0.162
BSA	0.09	0.402
BMI	0.07	0.499
Cumulative dose: Paclitaxel	0.08	0.465
Cumulative dose: Carboplatin	0.04	0.719

**Table 4:** Neurological examination findings of the study population.

Neurologic Findings (n, %)	Total n = 42	Normal Weight n = 24	Overweight n = 18	Adjusted *p*-Value	Cohen’s h	Calculated Power (%)
Allodynia	3 (7.14%)	2 (8.33%)	1 (5.56%)	1.000	0.110	6.4
Hyporeflexia	5 (11.90%)	4 (16.67%)	1 (5.56%)	0.495	0.365	21.6
Large fiber deficit	19 (45.24%)	7 (29.17%)	12 (66.67%)	0.111	0.770	69.4
Paresthesia	6 (14.29%)	5 (20.83%)	1 (5.56%)	0.495	0.472	32.8

*p*-values were calculated using Fisher’s exact test, and multiple comparisons were corrected using the Benjamini–Hochberg (BH) method to control the false discovery rate. Cohen’s h represents the effect size for differences in proportions between groups. Post hoc power analysis was conducted using the observed group sizes (normal weight: n = 24; overweight: n = 18), the observed effect sizes (Cohen’s h), and a two-sided alpha of 0.05. Calculated power (%) indicates the probability of detecting the observed effect size given the sample sizes. Low power (<80%) suggests a potential risk of Type II error for nonsignificant findings.

## Data Availability

The data that supports the findings of this study are available from the corresponding author upon reasonable request.

## Supplementary Materials

The following supporting information can be downloaded at: https://www.mdpi.com/article/10.3390/jcm14134485/s1, Figure S1: Correlation matrix of the laboratory findings. Kendall rank correlation coefficient (τ) was used to examine the relationships between variables; Table S1: Correlation between laboratory findings and Total Neuropathy Score (TNS). Kendall’s rank correlation coefficient (τ) was used for the associations with TNS; Table S2: Causal mediation analysis evaluating the role of BSA in the relationship between BMI and CIPN severity (measured by TNS); Table S3: Univariable linear regression analysis for possible confounders in the relationship between BMI and CIPN.

## Abbreviations

CIPN = Chemotherapy-induced peripheral neuropathy, BSA = Body surface area, BMI = Body mass index, ESR = Erythrocyte sedimentation rate, CRP = C-reactive protein, AST = Aspartate transaminase, ALT = Alanine transaminase, CA-125 = Cancer antigen 125, CEA = Carcinoembryonic antigen, CA-19.9 = Carbohydrate antigen 19.9, ANC = Absolute neutrophil count, total neuropathy score (TNS), Common Terminology Criteria for Adverse Events, or the patient-reported Functional Assessment of Cancer Therapy/Gynecologic Oncology Group-Neurotoxicity (FACT/GOG NTX), clinical version of the TNS (TNSc).
