# Supplementary material for: Higher Body Mass Index Is Related to Severe Chemotherapy-Induced Peripheral Neuropathy in Patients with Ovarian Cancer: A Preliminary Retrospective Study
Source: J Clin Med. 2025 Jun 25;14(13):4485. doi: 10.3390/jcm14134485 (PMC12249665; doi:10.3390/jcm14134485)
Supplement: Supplementary file 1 [file jcm-14-04485-s001.zip › jcm-3643589-supplementary.pdf]

Supplementary Figure S1. Correlation matrix of the laboratory findings. Kendall rank correlation coefficient (τ) was used to examine the relationships between variables.

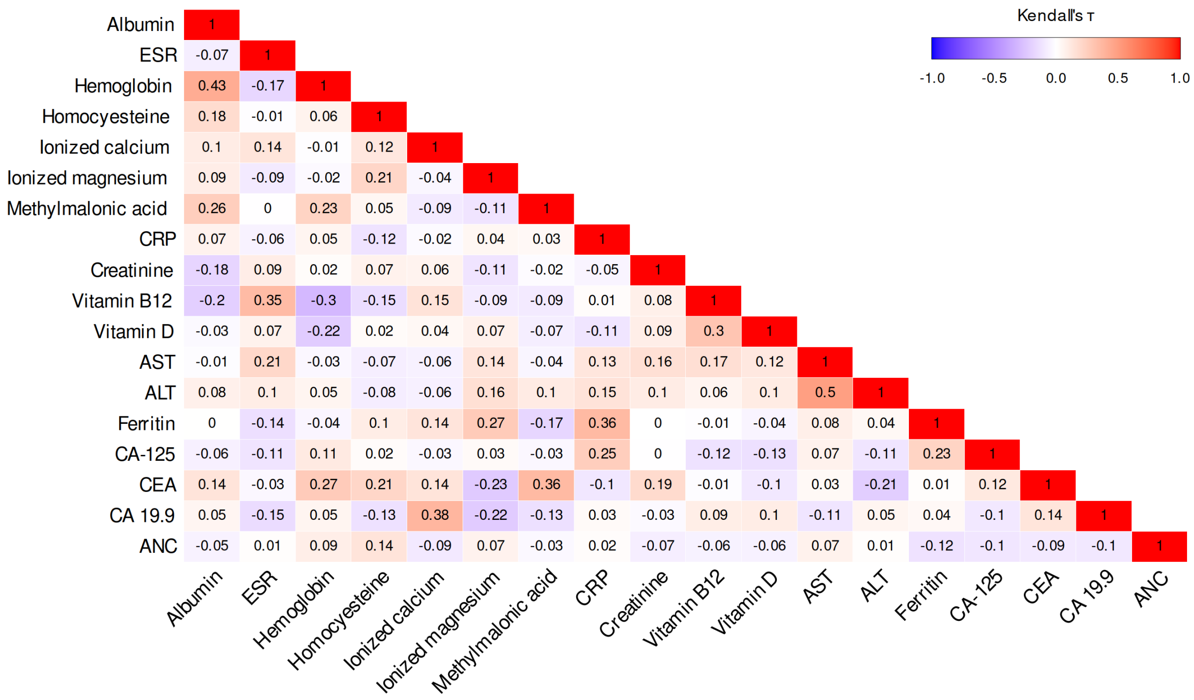

Supplementary Table S1. Correlation between laboratory findings and Total Neuropahty Score (TNS). Kendall's rank correlation coefficient (τ) was used the associations with TNS.

Laboratory findings	Kendall's rank correlation coefficient (τ) with TNS	P-value
Albumin	-0.01	0.912
ESR	0.10	0.371
Hemoglobin	-0.01	0.913
Homocyesteine	0.06	0.571
Ionized calcium	0.03	0.759
Ionized magnesium	-0.09	0.438
Methylmalonic acid	0.15	0.169
CRP	-0.14	0.218
Creatinine	-0.01	0.896
Vitamin B12	-0.09	0.414
Vitamin D	-0.05	0.627
AST	0.02	0.836
ALT	-0.14	0.213
Ferritin	0.00	0.975
CA-125	0.18	0.114
CEA	0.38	0.013
CA 19.9	-0.01	0.932
ANC	-0.07	0.520

Supplementary Table S2. Causal mediation analysis evaluating the role of BSA in the relationship between BMI and CIPN severity (measured by TNS).

Outcome	Effect	Estimate	95% CIL	95% CIH	p-value
TNS	Average Causal Mediation Effect	-1.183	-3.393	1.2	0.33
	Average Direct Effect	4.28	1.146	7.08	0.006
	Total Effect	3.097	0.68	5.43	0.02
	Proportion Mediated	-0.382	-2.172	0.44	0.346

Supplementary Table S3. Univariable linear regression analysis for possible confounders in the relationship between BMI and CIPN.

Variable	Coefficient for CIPN severity	P-value	Coefficient for CIPN onset time	P-value
BMI	0.189	0.332	-0.13	0.091
BMI group (Ref=normal weight)	3.097	0.02	-0.661	0.226
BSA	3.551	0.447	-3.055	0.097
Age	0.1	0.24	0.015	0.669
Type 2 DM	3.114	0.083	1.101	0.126
Hypertension	0.085	0.956	-0.274	0.658
